# Cancer stage and pack-years, but not p16 or HPV, are relevant for survival in hypopharyngeal and laryngeal squamous cell carcinomas

**DOI:** 10.1007/s00405-018-4997-1

**Published:** 2018-05-09

**Authors:** Valerie Dahm, Andrea Haitel, Alexandra Kaider, Isabella Stanisz, Andrea Beer, Claudia Lill

**Affiliations:** 1 Department of Otorhinolaryngology, Medical University of Vienna, Währinger Gürtel 18-20, 1090 Vienna, Austria; 2 Department of Pathology, Medical University of Vienna, Vienna, Austria; 3 Center for Medical Statistics, Informatics and Intelligent Systems, Medical University of Vienna, Vienna, Austria

**Keywords:** HPV, P16, Laryngeal carcinoma, Hypopharyngeal carcinoma, Squamous cell carcinoma

## Abstract

**Purpose:**

Recently, p16 has been included in the TNM guideline for oropharyngeal carcinomas. The role of HPV and p16 in hypopharyngeal and laryngeal carcinomas has not yet been established sufficiently.

**Methods:**

Hundred and thirty-four patients with hypopharyngeal and laryngeal carcinomas were included in this retrospective analysis. Only patients with known HPV status were eligible for the investigation. Survival probabilities were estimated for different risk factors.

**Results:**

Eighty-five patients presented with laryngeal carcinoma and 49 patients with hypopharyngeal carcinoma. 8% were HPV positive (10.6% laryngeal, 4.1% hypopharyngeal carcinoma). Median follow-up time was 58 months. We observed a significantly better overall survival for patients with an early tumor stage compared to advanced carcinoma. One of the hypopharyngeal HPV positive carcinomas was also p16 positive and one was p16 negative. Of the nine HPV positive laryngeal carcinomas, four were p16 positive and five p16 negative. Neither patients who were HPV positive nor patients positive for p16 showed a significantly better outcome than HPV or p16 negative patients. In contrast, nicotine pack-years showed a highly significant correlation with survival in our patient collective.

**Conclusions:**

The data suggest that tumor stage and nicotine exposure seem to have the highest impact on survival in hypopharyngeal and laryngeal squamous cell carcinoma patients. There is no evidence for a better survival for p16 positive or HPV positive patients with hypopharyngeal or laryngeal squamous cell carcinoma. HPV seems to play a minor role in these entities of head and neck carcinoma.

## Introduction

More than 30 years ago, a link between human papillomavirus (HPV) and head and neck cancer (HNC) was found. Since then numerous studies have been performed on this topic and showed a better prognosis for HPV positive oropharyngeal squamous cell carcinomas (OPSCC) than for HPV negative OPSCCs. This fact is underlined by the inclusion of p16 in the updated TNM classification [1]. The role of p16 and HPV in non-oropharyngeal squamous cell carcinomas has not yet been established sufficiently, varying results have been presented.

The aim of this study was to evaluate patients with HPV and/or p16 positive and negative hypopharyngeal and laryngeal squamous cell carcinomas and compare overall survival. Additionally, overall survival for traditional risk factors and tumour stage was reviewed.

## Materials and methods

Patients with hypopharyngeal and laryngeal carcinomas treated at the Medical University Hospital of Vienna between 1 January 2007 and 31 December 2016 were included in this retrospective analysis. Only patients with histologically proven squamous cell carcinoma and detected HPV status were eligible for the investigation. For all patients, pre-treatment biopsies were used for HPV and p16 detection. Patients’ age at diagnosis, tumour localization, TNM stage, p16 status, HPV status, nicotine and alcohol consumption, treatment modality and outcome were evaluated.

The study was conducted according to permission 1457/2017 from the Ethical Committee at the Medical University of Vienna, Austria.

### P16 and HPV detection

**Fig. 1:**
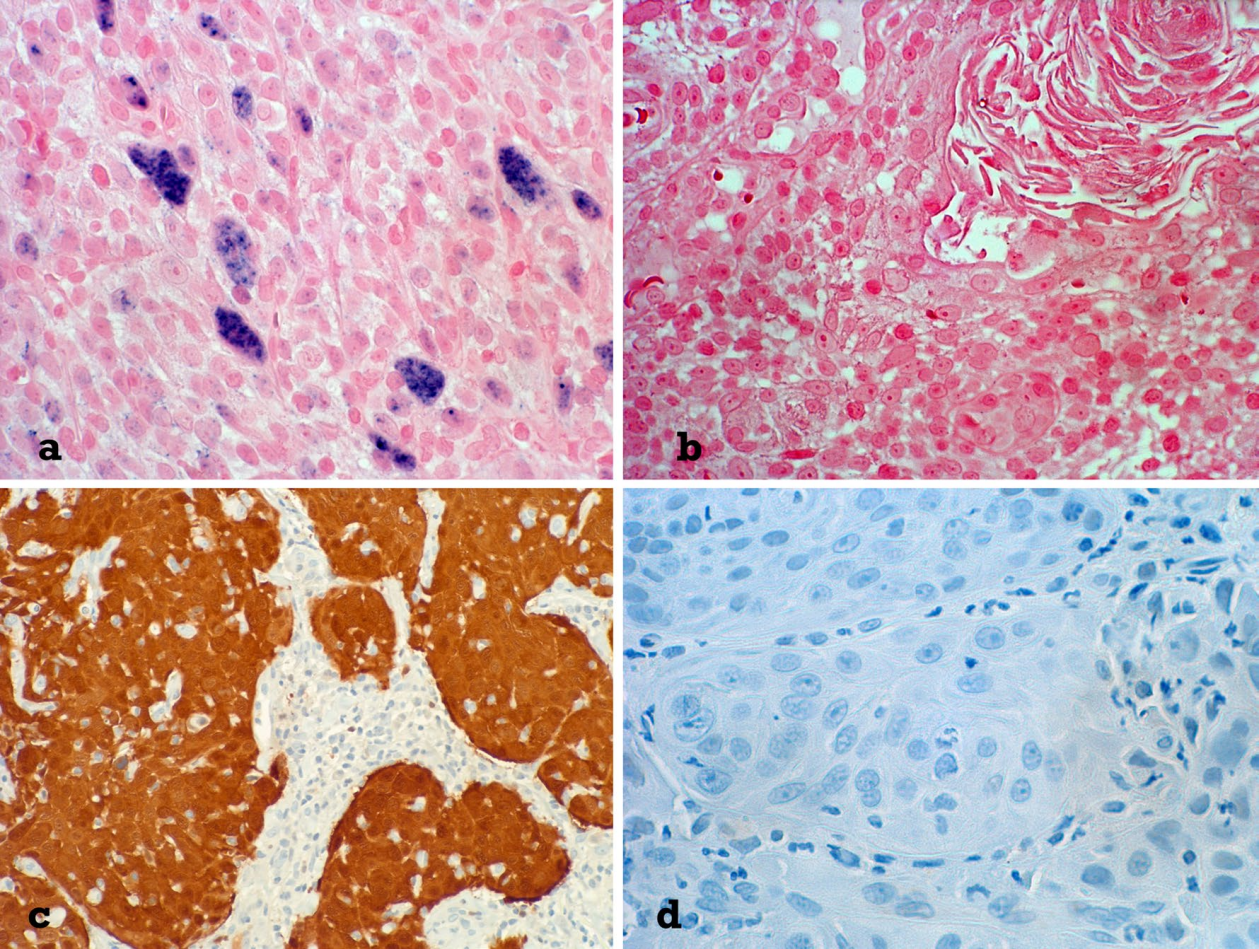
**a** Typical nuclear blue in situ hybridization reaction in HPV-associated squamous cell carcinoma (Original magnification 1:400). **b** Squamous cell carcinoma without in situ hybridization signal, representing HPV negative tumour (Original magnification 1:400). **c** HPV-associated squamous cell carcinoma with strong p16 positivity (Original magnification 1:400). **d** P16 negative squamous cell carcinoma (Original magnification 1:400)

Formalin fixed and paraffin embedded material of representative tumour blocks was sliced serially and 4 µm thick slides were deparaffinised and stained automatically by the use of BENCHMARK ULTRA (VENTANA). For HPV in situ hybridization INFORM HPV II Family 6 Probe (VENTANA, ready to use) and INFORM HPV III Family 16 Probe (B) (Ventana, ready to use) were incubated with pre-treatment CC2 (12 min, 86 °C) followed by ISH/VIEW Blue Plus Detection Kit (Fig. 1). Only a typical nuclear staining was rated positive according to the VENTANA HPV Interpretation guide ([2]).

For p16 immunohistochemistry CINtec p16 (VENTANA, ready to use) with pretreatment CC1 (36 min, 95 °C) and Ultra view Universal DAB Detection kit were used (Fig. 1). Interpretation was based on previous HPV detection guidelines [2].

### Definitions

Non-smokers were defined as patients who stated never to have smoked. Smokers were defined as patients who had smoked regularly before or were still active smokers. Patients were asked how many cigarettes they smoked and the duration of cigarette consumption. Pack years were then calculated from the given numbers. Patients who declared to drink alcohol on a regular or daily basis were defined as alcohol drinkers. Non-alcohol drinkers were defined as patients who never drank alcohol or only on vary rare occasions.

### Statistical analysis

The HPV prevalence is described by the percentage of HPV positive patients [with an exact 95% confidence interval (CI)]. The inverse Kaplan–Meier method [3] was used for calculation of the median follow-up time. Overall survival probabilities were estimated using the Kaplan–Meier method and the log-rank test was applied for statistical comparisons of survival curves. Univariate and multivariable Cox proportional hazards regression models were performed to evaluate the unadjusted and adjusted influence of HPV status, tumour stage, age, gender, alcohol consumption and smoking on overall survival. The Firth’s bias correction was used to avoid a bias due to the rather small number of events [4]. The hazard ratios (HR) with 95% confidence intervals are given to describe the strengths of the potential prognostic factors. Two-sided *p* values less than 0.05 are considered as indicating statistical significance. All analyses were performed using SAS software version 9.4 [SAS Institute Inc. (2002–2012); Cary, NC, USA].

## Results

### Patient characteristics

In total, 134 patients were included. Of all 134 patients, the following characteristics were known: patient age at time of diagnosis, TNM classification, treatment modality, HPV status, and tumour localization. P16 status, nicotine and alcohol consumption was not known in all patients. Median follow-up time was 58 months (range 0–104 months). Patients were treated with surgery, surgery and (chemo-/immuno-) radiotherapy or primary (chemo-/immuno-) radiotherapy. Two patients refused any treatment.

Patient characteristics are summarized in Tables 1 and 2. Of 134 patients, 11 were HPV positive, 2 hypopharyngeal carcinomas and 9 laryngeal carcinomas.

**Table 1:** Patient details divided into two groups: hypopharyngeal and laryngeal carcinomas

Carcinoma	Hypopharynx (49)	Larynx (85)	Total (134)
Patient characteristics			
Male	39 (80%)	71 (84%)	120 (90%)
Age (mean ± standard deviation)	58.4 (± 8.1)	61.5 (± 9.5)	60.4 (± 9.1)
HPV positive	2 (4%)	9 (11%)	11 (8%)
Tumour stage I–II	5 (10%)	44 (52%)	134
Tumour stage III–IVc	44 (90%)	41 (48%)	
Smokers	41 (84%)	63 (74%)	104

Tumour stage is presented according to UICC guidelines

**Table 2:** Patient tumour stages divided into two groups: hypopharyngeal and laryngeal carcinomas

Carcinoma localisation	Hypopharynx (*n* = 49)	Larynx (*n* = 85)	Total (*n* = 134)
Tumour stage			
Tumour stage T1	4 (14%)	29 (34%)	33 (24%)
Tumour stage T2	8 (16%)	29 (26%)	38 (28%)
Tumour stage T3	13 (27%)	15 (13%)	28 (21%)
Tumour stage T4	24 (49%)	12 (14%)	36 (27%)

Tumour stage is presented according to UICC guidelines

### Presence of p16 and HPV in hypopharyngeal and laryngeal carcinomas

Of the 134 patients included in the study, 85 patients had laryngeal carcinoma and 49 patients hypopharyngeal carcinoma. Nine patients were HPV high-risk positive (type 16) and two low-risk positive (type 6). In total, 11 of 134 patients were HPV positive [8.2%; 95% confidence interval (CI) (4.2–14.2%)].

Nine patients with laryngeal carcinoma and two patients with hypopharyngeal carcinoma were HPV positive (10.6 vs. 4.1%).

Of the nine HPV positive laryngeal carcinoma patients, seven were HPV high-risk positive and two low-risk positive. Of the two hypopharyngeal HPV positive carcinomas both were HPV high-risk positive.

In 81 cases p16 immunohistochemistry was available, 57 were p16 negative and 24 were p16 positive (70 vs. 30%). Of the 24 patients with p16 positive carcinomas 17 were laryngeal carcinomas and 7 hypopharyngeal carcinomas, respectively.

One of the hypopharyngeal HPV positive carcinomas was also p16 positive and one was p16 negative. Of the nine HPV positive laryngeal carcinomas four (44%) were p16 positive and five (56%) p16 negative. Both HPV low-risk positive carcinomas were p16 negative.

There was no significant difference between the two groups of patients (HPV positive and negative carcinomas). There was a similar distribution of gender, smoking as well as drinking habits and age (Table 3).

**Table 3:** Patient details divided into HPV positive and negative carcinomas. Tumour stage I or II (according to UICC guidelines) were summarized to an early tumour stage, tumour stage III to IVc (according to UICC guidelines) were summarized to an advanced tumour stage

Patient characteristics	HPV positive		HPV negative	
Patients (*n* = 134)	*n* = 11	Missing	*n* = 123	Missing
Male	8 (73%)	0	102 (83%)	0
Smoking	8 (73%)	0	96 (78%)	8 (7%)
Pack years [median (quartiles)]	40 (0–50)		40 (25–50)	
Alcohol	4 (36%)	1 (9%)	55 (45%)	13 (10.5%)
p16 positive	5 (45%)	0	19 (15%)	53 (43%)
Early tumour stage	2 (18%)	0	47 (38%)	0
Advanced tumour stage	9		76 (62%)	
Age mean (± standard deviation)	58.1 (± 9.6)		60.6 (± 9.1)	

### Survival analysis

All patients were included in survival analysis. In total, median survival for laryngeal carcinomas was 81 months and for hypopharyngeal carcinomas 25 months with a significantly better survival for laryngeal carcinomas (log-rank test: *p* < 0.001).

**Fig. 2:**
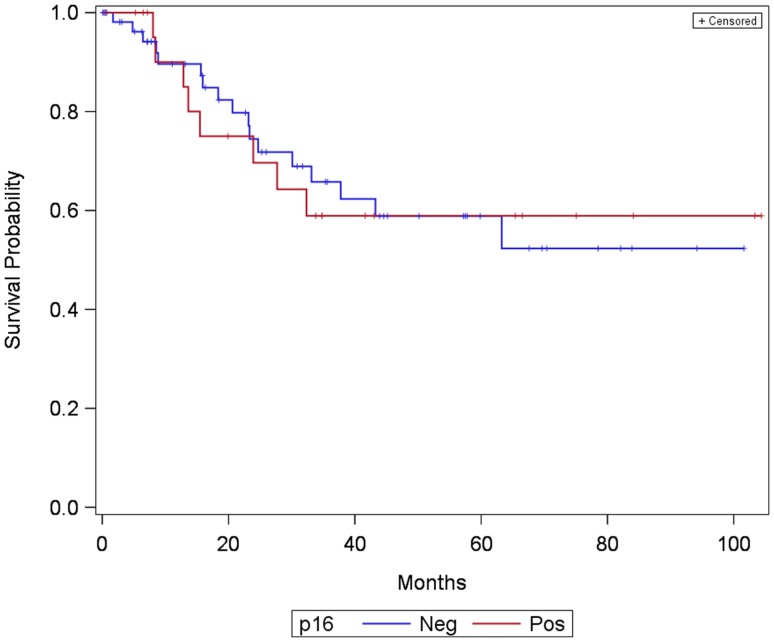
Kaplan–Meier curve showing survival of patients with laryngeal and hypopharyngeal carcinoma divided into two groups: p16 positive and p16 negative

Comparing overall survival for 81 patients with known p16 status, 24 p16 positive and 57 p16 negative, there was no significant impact on survival as shown in Fig. 2 (log-rank test: *p* = 0.956).

**Fig. 3:**
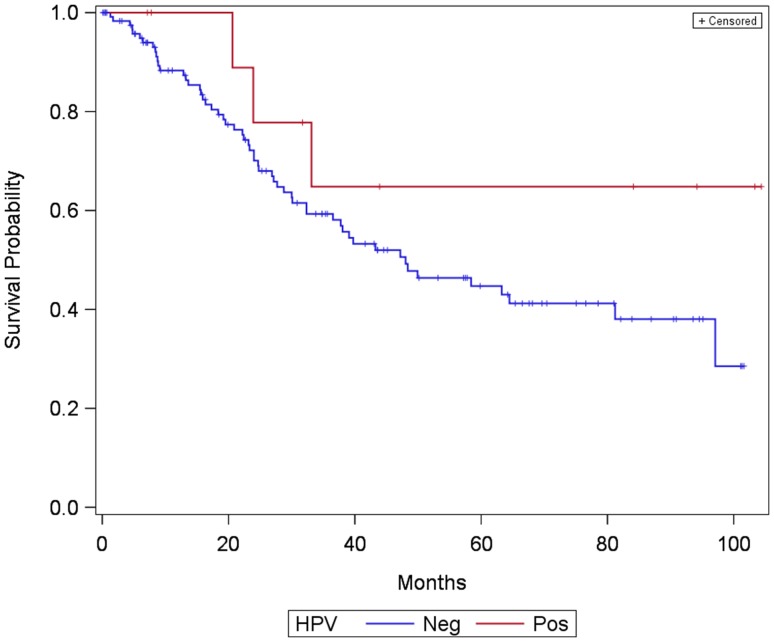
Kaplan–Meier curve showing survival of patients with laryngeal and hypopharyngeal carcinoma divided into two groups: HPV positive and HPV negative

Analysis of overall survival revealed that HPV positive and HPV negative carcinomas shows no significantly different overall survival rates [log-rank test: *p* = 0.175; HR = 0.53, 95% CI (0.14–1.35)] (Fig. 3).

Overall survival of patients with a tumour stage I or II (according to UICC guidelines)—summarized to an early tumour stage—showed a highly significant better overall survival than patients with a tumour stage III–IVc (according to UICC guidelines)—summarized to an advanced tumour stage (*p* < 0.001). Patients with an advanced tumour stage were four times more likely to die than patients with a lower tumour stage [HR = 4.2, 95% CI (2.19–8.98)].

**Fig. 4:**
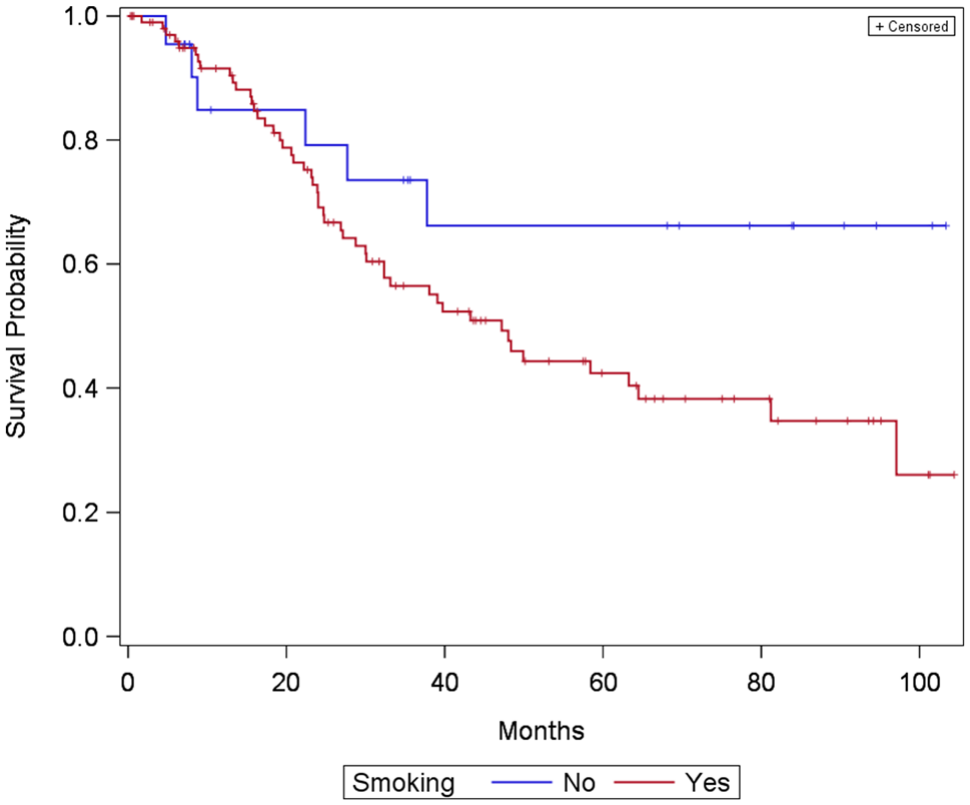
Kaplan –Meier curve showing survival of patients with laryngeal and hypopharyngeal carcinoma divided into two groups: smokers and non-smokers

Comparing smokers and non-smokers, the results showed a trend but reached no significance (*p* = 0.077) for overall survival of patients (Fig. 4). Nevertheless, analysis showed that with an increasing number of pack-years patients risk for dying increased significantly [HR = 1.01, 95% CI (1.00–1.02), *p* = 0.022].

Treatment options were divided into three subgroups. Group 1 consisting of patients undergoing surgery alone, group 2 included patients undergoing surgery followed by radiotherapy, chemoradiotherapy or immunoradiotherapy and finally group 3 included patients treated by primary radiotherapy, chemoradiotherapy or immunoradiotherapy. Patients in group 1 had a better outcome than patients in group 2 and both had a better outcome than patients in group 3. Comparison of all three groups reached a significant difference (*p* = 0.009).

Multivariable analysis revealed the prognostic factor tumour stage as the only statistically significant parameter on overall survival [HR = 4.32, 95% CI (2.11–9.95), *p* < 0.0001]. The influence of HPV was not statistically significant [HR = 0.42 95% CI (0.11–1.12), *p* = 0.087].

## Discussion

Tobacco and alcohol have been the most important risk factors for the development of head and neck carcinomas for the past decades. After studies including those of Fahkry et al., which could demonstrate that patients with HPV positive oropharyngeal carcinomas have at least half the risk of death than HPV negative patients [5], HPV has recently been acknowledged as a risk factor for oropharyngeal carcinoma by the International Agency for research on Cancer.

The role of p16 and HPV in hypopharyngeal and laryngeal carcinomas has not sufficiently been established yet. Especially in laryngeal and hypopharyngeal cancers HPV expression vary enormously. The prevalence of HPV DNA in laryngeal squamous cell carcinomas varies between 0 and 75% [6, 7]. HPV DNA positive hypopharyngeal squamous cell carcinomas have been described in 0–58% of cases [8]. Survival rates of HPV positive and negative tumours have been analysed by different research groups. Results have showed great variability. Dalianis et al. could show a significantly better overall survival for patients with hypopharyngeal carcinomas with combined HPV16 and p16 positive tumours compared to other patients with hypopharyngeal cancers (*p* = 0.0185). Joo et al. found a rate of 10.9% HPV positive hypopharyngeal carcinomas in 64 patients. In these patients there was a significant correlation of HPV high-risk positive carcinomas, younger age and non-smoking status. HPV-positive patients had a significantly better disease-free survival (*p* = 0.026) and disease-specific survival (*p* = 0.047) [9]. In a further study by Wendt et al. a trend for better overall survival in HPV-16 positive hypopharyngeal carcinomas (*p* = 0.031) could be found [10]. As supported by the results in our study, Meshman et al. and Fakhry et al. showed no improvement in overall survival of p16 positive hypopharyngeal or laryngeal squamous cell carcinoma patients [11].

The percentage of HPV positive carcinomas in our patient cohort (4.1% HPV positive hypopharyngeal carcinoma and 10.6% of laryngeal carcinomas) is similar to other studies [9, 10, 12]. Nevertheless, the prevalence of HPV seems to differ due to geographically different exposure. This phenomenon was analysed by Anantharam et al. The authors could show a higher prevalence rate of HPV in the US and a missing prevalence in South America in non-oropharyngeal SCC [13].

Another reason for variable HPV prevalence is the method of detection, where p16 plays an important role, as it is often determined as a surrogate marker for HPV status. P16 is a cyclin-dependent kinase inhibitor, which becomes up-regulated in HPV infected cells when E7 inactivates the tumour suppressor Retinoblastoma protein [11]. In oropharyngeal carcinomas p16 is widely used as reliable marker for HPV detection [14]. The hypothesis has been made that p16 could inhibit tumour invasion and therefore acts independently [15].

This hypothesis is supported by the results of studies by Meshman et al. or Chung et al. who had similar results to our study with a missing correlation of p16 and HPV [11, 16]. Of the 11 HPV positive laryngeal and hypopharyngeal carcinomas, in our study, only five were p16 positive and six were p16 negative, respectively. In contrast, both HPV positive hypopharyngeal carcinomas were p16 negative.

P16 might not be a good marker for HPV in non-oropharyngeal head and neck carcinomas.

Additionally, our results show that p16 expression and presence of HPV in laryngeal and hypopharyngeal carcinomas have no correlation to overall and disease-free survival. These results can also be compared to Wendt et al. [10], and Meshman et al. [11]. In contrast, Joo et al. observed a significantly better overall survival in hypopharyngeal SCCs, although the small number of patients presenting with hypopharyngeal carcinoma is always a limiting factor.

For normal laryngeal mucosa an incidence rate of HPV of up to 19% has been reported [17]. HPV can therefore be present without attributing to the existence of squamous cell carcinoma. A biologically active HPV infection needs to be associated with up-regulated protein p16 according to Torrente et al. Only HPV and p16 positive cases can be regarded as HPV-related cancer [17]. In this study, only five tumours were HPV and p16 positive, four of these tumours were laryngeal carcinomas. The number of HPV positive carcinomas which were also p16 positive was too small to perform further analysis.

Another important factor when analysing HPV and p16 positive tumours is the method of detection.

The golden standard for HPV detection has been discussed widely. At present, PCR is the most sensitive technique used in the search for viral genomes [18]. A positive result for HPV DNA PCR amplification only demonstrates the presence of HPV and does not necessarily imply its role in carcinogenesis [19].

In our study, HPV was detected routinely by in situ hybridization (ISH) as shown in Fig. 1. HPV ISH is known to be specific, but less sensitive than other HPV detection methods [14, 16, 20, 21]. In situ hybridization techniques permit identification of infections in which viral DNA is integrated into the host genome, revealing a nuclear pattern of staining [22]. If HPV is etiologic and has predictive value in laryngeal and hypopharyngeal squamous cell carcinomas remains unclear.

Not only the presence and detection of HPV play an important role, but also the type of HPV has an important impact. In total there are more than 100 known HPV subtypes. Kreimer et al. presented a systematic review of HPV types in HNSCCs, where HPV16 was the most common subtype in HPV positive HNSCCs. In oropharyngeal HNSCC HPV 18 was found less and more often in squamous cell carcinomas of the oral cavity and laryngeal carcinomas [23]. Other types of HPV were rarely or never detected in HNSCCs [23].

In our study, nine patients were HPV high-risk positive (HPV type 16) and two were HPV low-risk positive (HPV type 6), which corresponds to the results mentioned before.

Neither patients who were HPV positive nor patients positive for p16 showed a significantly better outcome than HPV or p16 negative patients.

In contrast, nicotine pack-years and tumour stage showed a highly significant correlation with survival in our patient collective. Correlation of tumour stage and survival in all carcinomas of the head and neck has been shown in several previous studies. Patients with a lower tobacco exposure were more likely to have a better prognosis than heavy smokers. Eight out of 11 HPV positive tumour patients were smokers and 10 out of 11 were also exposed to alcohol, which does not allow further analysis of HPV positive non-smokers and non-drinkers. In oropharyngeal carcinomas HPV positive patients often are non-smokers and do not or rarely consume alcohol. This fact cannot be transferred to laryngeal and hypopharyngeal carcinomas, whether in the present study or in our personal experience.

The survival results in this study depending on treatment options show that patients treated by surgery alone have a better outcome than patients treated by multiple modalities and patients treated by primary (chemo- or immuno-) radiotherapy. These results can be attributed to the fact that tumours with less extension are more often and easier treated by surgery alone and no further treatment is required. Patients with larger and/or inoperable tumours are treated with primary conservative treatment. These results therefore reflect the difference between early and advanced tumour stages rather than therapy outcomes.

## Conclusions

In conclusion, the data presented suggest that tumour stage and nicotine exposure seem to have the highest impact on survival in hypopharyngeal and laryngeal squamous cell carcinoma patients. We could not observe any sufficient correlation of p16 and HPV. There is no evidence for a better survival for p16 positive or HPV positive patients with hypopharyngeal or laryngeal squamous cell carcinoma. Active HPV infection seems to play a minor role in these non-oropharyngeal entities of head and neck carcinoma.
